# The Importance of Cl^−^ Exclusion and Vacuolar Cl^−^ Sequestration: Revisiting the Role of Cl^−^ Transport in Plant Salt Tolerance

**DOI:** 10.3389/fpls.2019.01418

**Published:** 2019-11-08

**Authors:** Honghong Wu, Zhaohu Li

**Affiliations:** ^1^College of Plant Science & Technology, Huazhong Agricultural University, Wuhan, China; ^2^College of Agronomy and Biotechnology, China Agricultural University, Beijing, China; ^3^Department of Botany and Plant Sciences, University of California, Riverside, CA, United States

**Keywords:** Cl^−^ exclusion, Cl^−^ transport, ion channels and transporters, salinity stress tolerance, vacuolar Cl^−^ sequestration

## Abstract

Salinity threatens agricultural production systems across the globe. While the major focus of plant researchers working in the field of salinity stress tolerance has always been on sodium and potassium, the transport patterns and physiological roles of Cl^−^ in plant salt stress responses are studied much less. In recent years, the role of Cl^−^ in plant salinity stress tolerance has been revisited and has received more attention. This review attempts to address the gap in knowledge of the role of Cl^−^ transport in plant salinity stress tolerance. Cl^−^ transport, Cl^−^ exclusion, vacuolar Cl^−^ sequestration, the specificity of mechanisms employed in different plant species to control shoot Cl^−^ accumulation, and the identity of channels and transporters involved in Cl^−^ transport in salt stressed plants are discussed. The importance of the electrochemical gradient across the tonoplast, for vacuolar Cl^−^ sequestration, is highlighted. The toxicity of Cl^−^ from CaCl_2_ is briefly reviewed separately to that of Cl^−^ from NaCl.

## Introduction

Soil salinity affects nearly 50% of all irrigated land in the world, and is a major constraint to crop yield (Fita et al., 2015). To meet the projected demand of feeding 9.3 billion people by 2050, global agricultural production must be increased by 60% from its 2005–2007 levels (van Ittersum et al., 2016). Therefore, understanding the mechanisms that underlie plant salt tolerance, especially its ion transport-related traits, is important, since it would allow the breeding of salt tolerant crops and thus mitigate the possible food shortage in the future.

Traditionally, adverse effects of soil salinity have been attributed to with Na^+^ toxicity, prompting the majority of studies on this topic (Munns and Tester, 2008; Horie et al., 2012; Deinlein et al., 2014; Maathuis, 2014; Wu et al., 2015; Hanin et al., 2016; Wu et al.,, 2018a). However, an increase in Na^+^ content (Munns and Tester, 2008; Wu, 2018) is always accompanied by Cl^–^ accumulation (Tavakkoli et al., 2010) and K^+^ loss (Wu et al., 2018b) in plants exposed to salt (NaCl) stress. K^+^ is the major inorganic nutrient cation in non-halophytes (Dreyer and Uozumi, 2011), and plays important roles in plant cell activities (Anschütz et al., 2014; Shabala and Pottosin, 2014; Wu et al., 2018c) and stress responses (Wang et al., 2013). Cl^−^ is a plant micronutrient and regulates leaf osmotic potential, and turgor, and stimulates growth in plants (Franco-Navarro et al., 2016). However high Cl^−^ solutions are toxic, and impair photosynthesis and growth (Tavakkoli et al., 2010; Tavakkoli et al., 2011). Specific reasons for these detrimental effects are much less understood than those of Na^+^, but the excessive accumulation of Cl^−^ in chloroplasts is one effect (Seemann and Critchley, 1985; Geilfus, 2018b).

In recent years, the role of Cl^−^ in plant salinity stress tolerance has attracted more attention. The role of Cl^−^ in halophytes was reviewed by (Bazihizina et al., 2019). They suggest that rather than targeting Cl^−^ exclusion, a better way to breed salt tolerant crops would be to improve the selectivity of the broadly selective anion-transporting proteins. Cl^−^ as an essential micronutrient and its beneficial role in plants (Raven, 2017; Wege et al., 2017), the role of Cl^−^ in organelle development (Geilfus, 2018a; Geilfus, 2018b), and control of Cl^−^ transport in plants (Li et al., 2017b) have been recently reviewed. Moreover, it is suggested nowadays that Cl^–^ is a beneficial macronutrient for plants (Franco-Navarro et al., 2016; Franco-Navarro et al., 2019). Unlike the above-mentioned recent reviews, the present mini review is focused on the main traits related to controlling Cl^−^ transport, and its role in plant salt tolerance.

## Cl^–^, a Largely Overlooked Ion in Plant Salt Tolerance

Cl^–^ is an essential micronutrient in plants. In some plant species e.g. soybean, and woody plants such as avocado, Cl^–^ showed more significant toxic effect than Na^+^ since they are better at excluding Na^+^ from the leaf blades than Cl^–^ (Munns and Tester, 2008). The toxicity threshold of Cl^–^ is estimated to be 15-50 and 4-7 mg per gram dry weight for Cl^–^ tolerant and sensitive species, respectively (Xu et al., 1999). Besides its roles in photosynthesis and in membrane potential stabilization, Cl^–^ also regulates enzyme activities in the cytoplasm and is involved in turgor and pH regulation (Teakle and Tyerman, 2010). Low Cl^–^ (< 5 mM) resulted in increased leaf area and biomass in tobacco plants (Franco-Navarro et al., 2016), whereas high Cl^–^ (> 120 mM) resulted in decreased biomass production in barley plants (Tavakkoli et al., 2011). Whereas high Cl^–^ (over 4 mg g^–1^ FW) shows toxicity due to interference with PSII quantum yield and photosynthetic electron transport rate (Tavakkoli et al., 2011; Carillo et al., 2019), Cl^−^ in a range of 0.2–2 mg g^–1^ FW (fresh weight) can function in the stabilization of the oxygen-evolving complex of photosystem II, in the maintenance of electrical potential in cell membranes, regulation of tonoplast H^+^-ATPase and enzyme activities (Marschner 1995; Broadly et al., 2012; Carillo et al., 2019). In tobacco, up to 4 mg g^–1^ FW, Cl^–^ can be important for the maintenance of water homeostasis (Franco-Navarro et al., 2016).

For most crop plants, Na^+^ is more toxic than Cl^–^. Salinity stress in some plant species, e.g. soybeans and woody plant species such as citrus, is due to more pronounced Cl^−^ toxicity (White and Broadley 2001). Such species are comparatively effective in excluding Na^+^, but do not prevent Cl^–^ from accumulating to toxic levels in leaves (Munns and Tester, 2008). It is known that Na^+^ toxicity is due to the similarity of the hydrated ionic radii of Na^+^ to that of K^+^, and is thus in competition with K^+^ for binding sites on enzymes that depend on K^+^ for activation. This is clearly not the case of Cl^–^. Antagonism between Cl^–^ and NO_3_^–^ can result in a reduction in the uptake and storage of nitrogen (Li et al., 2017b), which is the important source for protein synthesis and many metabolism products. At high concentrations of Cl^–^, limited nitrogen supply as a result of antagonism between Cl^–^ and NO_3_^–^ could be a reason for its toxicity in salinized plants. In the presence of high Cl^–^, a decrease of shoot NO_3_^–^ to Cl^–^ ratio is correlated with a significant shoot biomass reduction in *Arabidopsis*, and the overexpression *SLAH1* (a homologue of the slow type anion channel SLAC1) (Qiu et al., 2016) (Cubero-Font et al., 2016). Li et al. (2017b) recently proposed that the NO_3_^–^/Cl^–^ ratio might be used as a salt tolerance indicator, similar to the K^+^/Na^+^ ratio.

## CaCl_2_ Based Cl^–^ Toxicity in Ornamental and Horticultural Plant Species

Besides sodium salts, a wide range of other dissolved salts (e.g. those of Mg^2+^ and Ca^2+^ salts) are also present in saline soils (Tavakkoli et al., 2010). When reclaimed water is used for irrigation, (Wang et al., 2017), B^3+^, Ca^2+^, Cl^–^, Na^+^, and SO_4_^2–^ and their salts may be prevalent (Martinez and Clark, 2012; Nackley et al., 2015). In this context, there is potential for Cl^−^ toxicity to arise from CaCl_2_ instead of NaCl. Further, Cl^−^ toxicity may arise from the use of Cl^−^ salts, (such as KCl and CaCl_2_), as fertilizers. For example, to decrease the accumulation of nitrate in leafy vegetables, (to meet EU directives), calcium Cl^−^ is sometimes substituted for calcium nitrate (Carillo et al., 2019). Low level Ca^2+^ (below 10 mM) is known to ameliorate NaCl induced salinity stress symptoms in many plant species, e.g. *Arabidopsis* (Shabala et al., 2006), rice (Rahman et al., 2016), wheat (Al-Whaibi et al., 2012), barley (Cramer et al., 1991), and *Calligonum mongolicum* (Xu et al., 2017), etc. However, under moderate CaCl_2_ treatment (above 20 mM), greater Cl^–^ toxicity was observed in some ornamental, (e.g. *Callistemon citrinus* and *Viburnum lucidum*), and horticultural species, (e.g. *Ocimumu basilicum*), (Borghesi et al., 2013; Collaa et al., 2013; Borgognone et al., 2014; Carillo et al., 2019; Cirillo1 et al., 2019). These studies showed that CaCl_2_ salinity is able to induce ion imbalance and hyperosmotic stress even more severe than that of NaCl, strongly reducing plant growth and yield. These results can be partly attributed to the toxic effects of Cl^–^, (since CaCl_2_ has twice the Cl^−^ that the same number of moles of NaCl carries), for which the uptake and transport to leaves could be less controlled than that of Na^+^ (Collaa et al., 2013) in these studied ornamental and horticultural plant species.

## Channels and Transporters in Cl^–^ Transport

To date, the most commonly reported gene families for Cl^−^ channels are characterized as slow anion channels (SLAC channels), Cl^−^ channels (CLC), and aluminium activated malate transporters (ALMT transporters) (Skerrett and Tyerman, 1994). Seven CLCs (AtCLCa to AtCLCg, channels or transporters), localized in various intracellular membranes including the tonoplast (AtCLCa, AtCLCb, AtCLCc, and AtCLCg), thylakoid membrane (AtCLCe), and Golgi membrane (AtCLCd and AtCLCf), were found in *Arabidopsis* (Barbier-Brygoo et al., 2011; Herdean et al., 2016a). Other possible Cl^−^ candidate channels (including the putative one) are mechanosensitive channels (Maksaev and Haswell, 2012; Hamilton et al., 2015), AtVCCN1 (Herdean et al., 2016b), VvNRT1.4, and VvNAXT1 (Henderson et al., 2014). For Cl^−^ transporters, ALMT (aluminium-activated malate transporters) (De Angeli et al., 2013), CCC (cation chloride co-transporter) (Colmenero-Flores et al., 2007), DTX/MATE (detoxification efflux carrier/multidrug and toxic compound extrusion transporters) (Zhang et al., 2017), and AtNPF2 (Li et al., 2017b) are reported to be involved in Cl^−^ transport in salinized plants.

Besides Na^+^ toxicity, salinity (both Na^+^ and Cl^–^) also causes osmotic stress in plants. Cl^–^ influx has been observed in plant cells subjected to salinity (Shabala et al., 2005). Interestingly, hyperosmotic stress also induced net Cl^–^ uptake in bean mesophyll cells (Shabala et al., 2000). This leads to the question of whether the mechanosensitive channels might play a direct or indirect role in Cl^–^ transport in plant cells under salt stress. Falke et al. (1988) identified the first mechanosensitive Cl^−^ channel in tobacco protoplasts. Maksaev and Haswell (2012) found that MSL10 (mechanosensitive channel-like 10) shows a preference for Cl^–^ over Na^+^ (6:1) and might be involved in Cl^–^ efflux to relieve the membrane tension once the channel opens. MSL8 (Cl^–^ over Na^+^, 6.3:1) was also found to sense and respond to changes in *Arabidopsis* pollen hydration, and also to germination associated membrane tension (Hamilton et al., 2015). The role of mechanosensitive channels in Cl^–^ transport in plant salt stress responses should be studied in greater detail.

**Figure 1:**
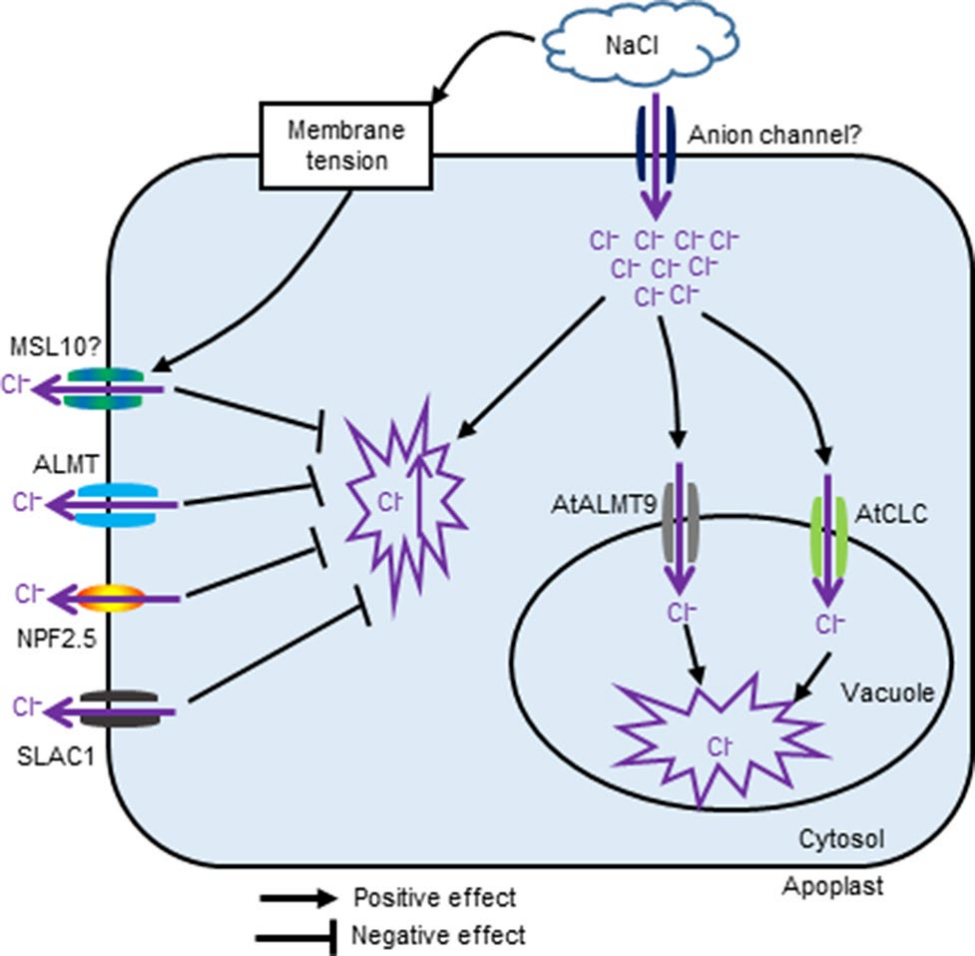
The proposed schematic showing channels and transporters involved in Cl^−^ transport in plants under salinity stress.

The channels and transporters involved in Cl^−^ transport in plants under salt stress are summarized in **Figure 1**. Interesting topics with a potential for future study include dissecting the possible role and contributions of Cl^–^ transport in plant overall salt tolerance, identifying specific channels/transporters involved in Cl^–^ transport, and establishing which contribute to salt tolerance, as well as investigating a possible link to other signalling events e.g. Ca^2+^ and ROS waves.

## Regulation of Cl^–^ Transport in Plant Salinity Stress Response

### Controlling Shoot Cl^–^ Accumulation: The Role of Root and Shoot Cl^–^ Exclusion Differs Between Plant Species

Cl^−^ in soil presents mainly as Cl^–^, and its movement within the soil is largely determined by water flows (White and Broadley, 2001). Compared to the advanced study of Na^+^ transport, much less data is available for Cl^–^ transport in plants under salinity. Teakle and Tyerman (2010) summarized several traits of Cl^–^ movement related to plant salt tolerance: 1) reduced net Cl^–^ uptake by roots, 2) reduced net xylem loading of Cl^–^, 3) intercellular compartmentation of Cl^–^, 4) intracellular compartmentation of Cl^–^, and 5) phloem recirculation and translocation within the plant. Efficient exclusion of Cl^–^ from either roots or shoots could avoid excessive accumulation of Cl^–^ in plant tissues.

A significantly lower shoot Cl^–^ concentration was found in salt tolerant barley varieties than in sensitive ones (Tavakkoli et al., 2011), suggesting that controlling the accumulation of shoot Cl^–^ is important for overall salt tolerance. However, whether this is due to root Cl^–^ exclusion or shoot Cl^–^ exclusion still needs to be answered. A question also remains over the differences that may exist between plant species. Chen et al. (2013) showed that wild type soybean has stronger root Cl^–^ exclusion (efflux) than the relatively salt sensitive cultivated soybean varieties, suggesting the important role of root Cl^–^ exclusion in soybean salt tolerance. In citrus rootstocks, tolerance in salt tolerant species, which have significantly lower shoot Cl^–^, is mostly conferred by superior root resistance to Cl^–^ uptake (Moya et al., 2003). This is further demonstrated by Brumos et al. (2010). In barley, to the contrary, less accumulation of shoot Cl^–^ in salt tolerant than sensitive species (Tavakkoli et al., 2011) may not be due to root Cl^–^ exclusion. It has been shown that although cytosolic and vacuolar Cl^–^ concentration is not different between salt tolerant and sensitive barley varieties, a 73% higher (*P < 0.001*) apoplast Cl^–^ in root cortical cells was found in salt sensitive compared to tolerant barley varieties (Flowers and Hajibagheri, 2001). Also, a strong and positive correlation was found between grain yield and transcripts of *HvSLAH1* and *HvSLAC1*, (anion channels that mediate Cl^−^ efflux), in barley leaves (Liu et al., 2014). This suggests that shoot Cl^–^ exclusion in barley is associated with overall salt tolerance. Shoot Cl^−^ exclusion is also associated with salt tolerance in some other species, e.g. grapevine, *Lotus*, and *Glycine* etc. (Teakle and Tyerman, 2010). Significantly lower xylem Cl^–^ was found in relatively salt tolerant varieties compared to salt sensitive varieties of durum wheat (Line 149 vs Tamaroi) (Läuchli et al., 2008), and *Lotus corniculatus* (*L. tenuis* vs *L. corniculatus*) (Teakle et al., 2007). Overall, the mechanisms controlling shoot Cl^–^ accumulation evidently do differ between plant species.

### Shoot and Root Cl^–^ Exclusion: Which Channels or Transporters Play the Major Roles?

To date, the topic of key transporters and channels involved in shoot and root Cl^−^ exclusion is still not yet fully explored. In terms of breeding salt tolerant species, knowing the key transporters and channels controlling Cl^−^ exclusion could be very helpful. For example, overexpression of the genes *SOS1*, (encoding a key Na^+^/H^+^ antiporter for Na^+^ extrusion from cytosol to apoplast), (Yang et al., 2009; Yue et al., 2012), and *NHX1*, (encoding a key K^+^, Na^+^/H^+^ exchanger for sequestrating Na^+^ from cytosol to vacuole), (Apse et al., 1999; Zhang and Blumwald, 2001; Chen et al., 2007; Gouiaa et al., 2012), resulted in increased overall salt tolerance in many plant species. Pyramiding with the approach proposed by Bazihizina et al. (2019), (that of improving the selectivity of the broadly selective anion-transporting proteins), may yield enhanced Cl^−^ exclusion ability and be an important strategy for breeding salt tolerant crops.

SLAC1, a channel located in the plasma membrane, is highly permeable to malate and Cl^−^ (Negi et al., 2008). Transgenic overexpression of *AtSLAC1* in tobacco BY-2 cells enhanced cryptogein (an elicitor able to induce ROS production)-induced Cl^−^ efflux (Kurusu et al., 2013). Plants with a mutation in *slac1*, (the gene for SLAC1 which mediates Cl^−^ efflux), also showed increased Cl^−^ content in guard cells (Negi et al., 2008). Together, these reports suggest that SLAC1 is an anion channel which is involved in Cl^−^ exclusion. SLAH1, a homologue of SLAC1, modulates shoot Cl^−^ accumulation, and thus salt tolerance, in *Arabidopsis* (Cubero-Font et al., 2016; Qiu et al., 2016). It has been shown that the upregulation of the genes *HvSLAH1* and *HvSLAC1* in leaves is linked with higher barley grain yield in the field (Liu et al., 2014).

Besides SLAC1, CLC channels/transporters and ALMT transporters might also play a role in Cl^−^ exclusion. Herdean et al. (2016a) proposed that the thylakoid Cl^−^ channel AtCLCe functions in Cl^−^ homeostasis. Wei et al. (2016) showed that by regulating Cl^−^ accumulation, the GmCLC1 channel confers enhanced salt tolerance in soybean. The ALMT (aluminium activated malate transporter) protein family is unique to plants and is able to mediate anion fluxes across cellular membranes (Barbier-Brygoo et al., 2011). At high external Cl^−^, the permeability ratio of TaALMT1, (Piñeros et al., 2008), and ZmALMT2 (Ligaba et al., 2012), towards malate and Cl^−^ is around 1, suggesting that these channels might be able to mediate a substantial amount of Cl^−^ efflux. AtALMT12, a plasma membrane targeted anion transporter, is mainly permeable to Cl^−^ and nitrate and is involved in stomatal closure (Meyer et al., 2010). Furthermore, Li et al. (2017a) found that AtNPF2.5 (NRT1/PTR Family protein), a Cl^–^ permeable transporter predominantly expressed in root cortical cells, modulates Cl^−^ efflux from roots. Mutation of the *AtNPF2*.5 gene in *Arabidopsis* resulted in impaired root exclusion and thus a higher shoot Cl^–^ accumulation (Li et al., 2017a). Overexpression of this gene might increase root Cl^–^ exclusion and thus may allow enhancement of overall salt tolerance in plants. Further, besides its role in limiting shoot Na^+^ accumulation, the endoplasmic reticulum located transporter GmSALT3 (encoded by “salt tolerance-associated gene on chromosome 3”) (Guan et al., 2014) was found to be involved in leaf Cl^−^ exclusion in soybean under salinity stress (Liu et al., 2016). Moreover, the possibility of involvement of the CCC (cation chloride co-transporter) family in Cl^−^ exclusion cannot be completely ruled out. For example, a *ccc2* mutant showed significant higher shoot Cl^−^ than wild type Arabidopsis, and shoot Cl^−^ content was significantly reduced in the *ccc2* lines expressing *VviCCC* under salt stress, thus suggesting its role in shoot Cl^−^ exclusion (Henderson et al., 2015). Overall, the current literature suggests that, among the above-mentioned Cl^−^ channels and transporters, SLAC1 and NPF2.5 are most likely to play an important role in shoot and root Cl^−^ exclusion.

### Vacuolar Cl^–^ Sequestration: Another Possible Component for Plant Salt Stress Tolerance?

The vacuole is an organelle that occupies up to 90% of the volume of plant cells (Gao et al., 2015). Depending on the plant species, cell type, and measurement method, reports of electrical difference across the tonoplast vary from -31 to +50 mV (Ginsburg and Ginzburg, 1974; Mertz and Higinbotham, 1976; Rona et al., 1980; Kikuyama, 1986; Miller et al., 2001; Wang et al., 2015). This electrochemical gradient across the tonoplast could possibly facilitate movement of Cl^–^ from the cytosol to the vacuole through channels or transporters, even if vacuolar Cl^–^ is higher than that of the cytosol. Furthermore, the rise of Ca^2+^ has been seen to cause a transient change in both the plasma membrane and tonoplast potentials in *Nitella* (Kikuyama, 1986). Furthermore, tonoplast potential is shown to be +9 in barley root cells treated with 0.5 mM CaSO_4_, and +35 mV when treated with 1 mM KCl + 0.5 mM CaSO_4_ (Mertz and Higinbotham, 1976), thus demonstrating the variability of tonoplast potential according to media. Under saline conditions, the tonoplast potential could be changed to be more negative or positive. Tonoplast potential in barley leaf mesophyll cells grown in non-saline conditions has been reported to be -4 mV, whereas it waxed negatively to -7 mV in barley plants subjected to salinity, (although this is still close to 0) (Cuin et al., 2003). It is thus shown that a more negative tonoplast potential exists in salt grown plants, which would allow for a more efficient active transport of Cl^−^ from the cytosol into the vacuole. The changes of tonoplast membrane potential induced by salinity may vary significantly between plant species, and cell types. A direct measurement of tonoplast potential in salt grown plants, of a species that is more intolerant to Cl^–^ than Na^+^, would help answer the question of the importance of vacuolar Cl^–^ in overall salt tolerance. If tonoplast potential is around zero in plants under salt stress which means the driven force for operating active transport is limited, then the movement of Cl^−^ will be predominantly dependent on the Cl^−^ gradient between the cytosol and vacuole, which may not allow the vacuole to sequestrate a large amount of Cl^−^. In terms of Cl^–^ vacuolar sequestration, after salt stress onset a certain time points (the time point could be varied in plant species and cell types), vacuolar Cl^–^ concentration is higher than cytosol and thus the ion channels with the nature of “downhill” activity will not be able to help in the vacuolar sequestration process. In this case, vacuolar sequestration of Cl^–^ is not likely to happen over a considerable period of time and is thus hardly an important component for plant salt tolerance. This challenges the proposed hypothesis that plants can store Cl^–^ in vacuoles to maintain cytosolic ion homeostasis, since in the above case, without the active transport of Cl^–^, it cannot be moved from the cytosol to vacuole against the proposed Cl^–^ gradient (high Cl^–^ stored in vacuole). Possibly, with the high cost of ATP, Cl^−^ transporters might play a role in this process. With tens of negative or positive tonoplast potentials, the expense on ATP for active Cl^–^ transport by Cl^−^ transporters can be significantly reduced and thus can allow plants to have a longer time period of vacuolar Cl^–^ sequestration under salt stress. We argue here that if some plant species have tens of negative or positive tonoplast potentials under saline conditions, vacuolar Cl^–^ sequestration could be one of the components of overall salt tolerance. Further studies are required to answer the above questions.

The location of AtCLCa, AtCLCb, AtCLCc, AtCLCg, and AtALMT9 Cl^−^ channels and transporters are known in the tonoplast, (De Angeli et al., 2013; Wei et al., 2015), and hints at the possible contribution of the vacuolar sequestration of Cl^-^ to salt tolerance in plants. Overexpression of the tonoplast located *AtCLCc* results in higher Cl^−^ accumulation and increased overall salt tolerance in the overexpression line in wild type Arabidopsis (Nguyen et al., 2016; Hu et al., 2017). Also, overexpression of *GmCLC1* in soybean hair roots results in more Cl^−^ sequestration in roots and thus less Cl^−^ being transported to the soybean shoot (Wei et al., 2016). AtALMT9, (a malate-activated Cl^−^ channel located in the tonoplast), is involved in vacuolar Cl^–^ sequestration and is required for stomatal opening (De Angeli et al., 2013). Furthermore, *AtALMT9* is transcriptionally up-regulated under salt stress, and *almt9* knockout mutants have reduced shoot accumulation of Cl^−^ (Baetz et al., 2016). Altogether, the above results suggest that vacuolar Cl^–^ sequestration might be another component for overall plant salt tolerance. However, large scale experiments are still needed to validate this supposition.

## Inconsistent Results of Cl^–^ Content in Plant Species With Contrasting Salinity Tolerance: the Importance of Intracellular Distribution of Cl^–^ in Plant Salt Tolerance

It has been argued that plant salt tolerance is related to the ability to regulate both Na^+^ and Cl^–^ transport to avoid toxicity (Tavakkoli et al., 2011). Under salinity stress, the most tolerant *Brassica* species showed less accumulation of Cl^–^ in the leaf than the sensitive one (Chakraborty et al., 2016). Interestingly, a significantly lower shoot Cl^–^ concentration was found in the salt sensitive bread wheat variety Krichuaff, than in the tolerant variety Berkut, under mild saline conditions (Genc et al., 2010). Also, Cl^–^ concentration in the xylem sap extracted from cut stems is significantly lower in melon, (which is relatively salt sensitive), than in pumpkin grafted on melon (which is relatively salt tolerant) (Edelstein et al., 2011). With the knockout of the vacuolar Cl^–^ channel ALMT9, which is highly expressed in the vasculature of shoots and roots, a reduced accumulation of Cl^-^ in the shoot was observed, showing that vacuolar Cl^–^ loading is crucial in controlling whole-plant ion movement during exposure to salinity (Baetz et al., 2016). The inconsistent relationship between Cl^-^ accumulation and genotypic salt tolerance suggests that the intracellular distribution of Cl^–^ is also important for plant salt tolerance. This is again in support of the viewpoint that vacuolar sequestration of Cl^–^ is important in salt tolerance of plants.

## Conclusion

Compared with well-studied Na^+^ and K^+^, knowledge on Cl^–^ in plant responses to salt stress is relatively limited. In this review, we argue that vacuolar Cl^–^ sequestration could play a role in salt tolerance, at least in plant species that have either positive or negative tonoplast potential amounting to tens of mV. We also suggest that the key channels/transporters of Cl^–^ exclusion in plants under saline condition still need to be investigated. The restriction of shoot Cl^–^ accumulation, both by root and/or shoot Cl^–^ exclusion, in different plant species is discussed in this review. Further, the toxicity of Cl^–^ originating from CaCl_2_, (which is particularly relevant to horticultural crops and ornamental plants), is briefly reviewed, and compared with the toxicity of Cl^–^ from NaCl.

## Author Contributions

HW and ZL wrote the manuscript.

## Funding

This work was supported by funding from Huazhong Agricultural University (11041910138) to HW and NSFC funding to ZL and to HW (31901464).

## Conflict of Interest

The authors declare that the research was conducted in the absence of any commercial or financial relationships that could be construed as a potential conflict of interest.
